
JOURNAL INFORMATION
==============================
NLM Title Abbreviation: ScientificWorldJournal
Journal ID: ScientificWorldJournal
NLM Journal ID: 101131163
Journal ID: TSWJ

The Scientific World Journal

ISSN: 2356-6140
EISSN: 1537-744X
Publisher: Wiley

ARTICLE INFORMATION
==============================
PMCID: PMC6084643
PMID: 30147544
DOI: 10.1100/tsw.2001.110
Article ID: 836757
Article version: 1
Subjects: Short Report

B Cell Precursors in Senescent Mice Exhibit Decreased Mitotic Recruitment, Increased Apoptosis, and Altered Expression of Bcl-2 Family Members

Riley Richard L. *
Sherwood Erin M.
Department of Microbiology and Immunology, University of Miami School of Medicine, P.O. Box 016960 (R-138), Miami, FL 33101, USA
*Richard L. Riley: rriley@med.miami.edu
Publication date: 2001
Electronic publication date: 2001 Oct 3
Volume: 1
First page: 70
Last page: 70
Copyright: Copyright © 2001 Richard L. Riley and Erin M. Sherwood.
Copyright year: 2001
License: This is an open access article distributed under the Creative Commons Attribution License, which permits unrestricted use, distribution, and reproduction in any medium, provided the original work is properly cited.
License URL: https://creativecommons.org/licenses/by/3.0/

Funding: National Institutes of Health AG 15474

==============================
Miami *Nature Biotechnology* Short Reports
*TheScientificWorld* (2001) 1(S3), 70SR
ISSN 1532-2246; DOI 10.1100/TSW.2001.110

### B CELL PRECURSORS IN SENESCENT MICE EXHIBIT DECREASED MITOTIC RECRUITMENT, INCREASED APOPTOSIS, AND ALTERED EXPRESSION OF BCL-2 FAMILY MEMBERS

Richard L. Riley* and Erin M. Sherwood

Department of Microbiology and Immunology, University of Miami School of Medicine,
P.O. Box 016960 (R-138), Miami, FL 33101
* rriley@med.miami.edu

**INTRODUCTION.** Senescence in inbred strains of mice affects lymphopoiesis within the bone marrow (1,2). In the development of B lineage cells, old age is associated with a significant decline in pre-B cells. This could result from increased cell death (apoptosis) and/or decreased mitotic activity. We have determined the mitotic activity of B cell precursors from aged mice *ex vivo* and the kinetics of aged pro-B cell proliferation *in vitro* at the single-cell level in response to IL-7. Furthermore, we have assessed susceptibility of *ex vivo* aged B cell precursors to apoptosis and expression of the survival molecules Bcl-$x_L$ and Bcl-2 and the pro-apoptotic molecule Baxa *in vitro*.

**METHODS.** Senescent (18-27 mo. old) and young adult (2-8 mo. old) BALB/c mice were obtained from the National Institute of Aging colony. Mitotic activity among *ex vivo* B cell precursors was assessed by fluorescence flow cytometric DNA cell cycle analysis. Apoptosis among *ex vivo* B cell precursors was measured with time during short-term (0-4 hr.) culture by flow cytometry (3). Subpopulations of pro-B and pre-B cells (Hardy Fractions A, B, C, C', D [4]) were determined by 4-color flow cytometry. B cell precursors were expanded *in vitro* upon stimulation with rmIL-7 for one week. *In vitro* proliferation was monitored using CFSE staining. Bcl-$x_L$, Bcl-2, and Baxa proteins were determined by Western blot.

**RESULTS.** *Ex vivo*, pro-B/early pre-B cells from aged mice exhibited normal proportions in S + G2/M active cell cycle stages. *Ex vivo* pro-B cells (Hardy Fractions A, B) and pre-B cells (Hardy Fractions C, C', D) only exhibited enhanced apoptosis upon short-term culture in mice with very severe deficits in B lymphopoiesis. *In vitro*, aged pro-B cells generally showed limited expansion in response to IL-7 when compared to young pro-B cells. CFSE-labeling indicated that pro-B cells from aged mice underwent similar numbers of cell divisions over time, as did young pro-B cells; however, the numbers of expanded pro-B cells was decreased. The expansion of aged pro-B cells in response to IL–7 *in vitro* also correlated inversely with the ratio of the pro-apoptotic protein Baxa to the survival protein Bcl-2. Expansion of aged pro-B cells was favored by particularly low Baxa:Bcl-2 protein ratios. The survival protein Bcl-$x_L$ was reduced in pro-B cells from the majority of aged mice, but this did not correlate with pro-B cell recovery *in vitro*.

**DISCUSSION.** Decreased pre-B cells *in vivo* and limited expansion of aged pro-B cells *in vitro* did not reflect poor proliferative capacity *per se*. More likely, aged mice have a lower frequency of pro-B/early pre-B cells, which are capable of undergoing extensive proliferation.

A subset of aged mice with severe deficits in B lymphopoiesis exhibited increased susceptibility to apoptosis among their B cell precursors. Altered expression of both pro- and anti-apoptotic molecules was observed in aged B cell precursors. Expansion of aged pro-B cells may be dictated by both recruitment of B cell precursors into mitosis and their survival.

**ACKNOWLEDGEMENTS.** Supported by NIH grant AG 15474 to RLR.

**REFERENCES.**

1. Riley, R.L., Kruger, M.G., and Elia, J. (1991) Clin. Immunol. Immunopathol. 59, 301-313
2. Sherwood, E.M., Blomberg, B.B., Xu, W., Warner, C.A., and Riley, R.L. (1998) J. Immunol. 161, 4472- 4476
3. Lu, L., Chaudhury, P., and Osmond, D.G. (1999) J. Immunol. 162, 1931-1940
4. Hardy, R.R., Carmack, C.E., Shinton, S.A., Kemp, J.D., and Hayakawa, K. (1991) J. Exp. Med. 173, 1213-1225
